# Artificial Saliva in Diabetic Xerostomia (ASDIX): Double Blind Trial of Aldiamed^®^ Versus Placebo

**DOI:** 10.3390/jcm9072196

**Published:** 2020-07-11

**Authors:** Bruna Sinjari, Beatrice Feragalli, Umberto Cornelli, Giovanni Belcaro, Ester Vitacolonna, Manlio Santilli, Imena Rexhepi, Gianmaria D’Addazio, Francesca Zuccari, Sergio Caputi

**Affiliations:** 1 Department of Medical, Oral and Biotechnological Sciences, University “G. d’Annunzio” of Chieti-Pescara, 66100 Chieti, Italy; b.feragalli@unich.it (B.F.); santilliman@gmail.com (M.S.); imena.rexhepi@gmail.com (I.R.); gianmariad@gmail.com (G.D.); scaputi@unich.it (S.C.); 2 Stritch School of Medicine, Loyola University Chicago, Maywood, IL 60611, USA; umbertocornelli@cornelliconsulting.it; 3 Irwin Labs, University of Chieti, 65010 Spoltore, Italy; cardres@abol.it; 4 Department of Medicine and Aging Sciences, University “G. d’Annunzio” of Chieti-Pescara, 66100 Chieti, Italy; ester.vitacolonna@unich.it; 5 Marchegiani Analysis Laboratory, 65122 Pescara, Italy; laboratorio.marchegiani@virgilio.it

**Keywords:** xerostomia, salivary flow, antioxidant capacity of saliva, aldiamed^®^

## Abstract

Xerostomia is a symptom frequently present in patients with type 1 (T1DM) and type 2 diabetes mellitus (T2DM). In the present trial, the activity of an artificial saliva (aldiamed^®^ spray) in comparison to a placebo spray were used to evaluate the xerostomia and the saliva antioxidant capacity (SAT). Sixty patients of both genders with T1DM or T2DM were randomized into two groups of 30 subjects each. The experiment was a double-blind study approved by the Ethics Committee of the “G. d’Annunzio University” of Chieti and Pescara. Moreover, measurements of the stimulated saliva flow rate and the ultrasonography of the submandibular and parotid glands were performed at both the study time points. The results demonstrated statistically significant differences between the treatments in terms of the xerostomia average score. Specifically, the values were at baseline and after 30 days 2.9 ± 1.31 and 3.0 ± 1.44 and 1.4 ± 1.48 and 2.4 ± 0.99 for aldiamed^®^ spray and the placebo, respectively. Meanwhile, no statistically significant differences were shown between the two groups for the other variables, such as the salivary flow rate, the antioxidant capacity of the saliva, and the ultrasonography of the major salivary glands.

## 1. Introduction

Diabetes mellitus (DM) is a metabolic disease due to a condition of chronic hyperglycemia [1]. Nowadays, DM has become a global epidemic disease, whose complications affect the life quality of sufferers [2]. The state of chronic hyperglycemia leads to various complications in numerous areas of the body, including the oral cavity [3]. Previous findings have reported that the oral complications of DM are due to impaired neutrophil function; elevated collagenase activity; the deficiency of collagen synthesis; and neuropathy, metabolic, and hemodynamic changes that primarily affect endothelial cells [4]. Oral manifestations related to DM include dry mouth (xerostomia), tooth caries, periodontal disease, oral candidiasis, burning mouth syndrome (BMS), altered taste, oral lichen planus, recurrent aphthous stomatitis, alteration in wound healing, increased tendency for infections, and salivary gland dysfunction [4]. Among the abovementioned oral complications of DM producing negative implications on quality of life, we can find the dry mouth symptom. People with diabetes are commonly affected by salivary gland dysfunction, which results in a reduction in salivary flow and an impairment in the composition of saliva [5]. In general, there are two different conditions reported in the literature and related to dry mouth [6]. The first phenomenon is the objective reduction in salivary flow called hyposalivation. This is defined as an unstimulated saliva flow rate of <0.1 mL/min, and it has been reported that 20% of people of all ages are affected, while the prevalence decreases to 3% when assessing the stimulated flow rate of saliva (<0.7 mL/min) [7]. The second condition often referred to as “dry mouth” is xerostomia, which refers to the patient’s subjective experience of a dry mouth. Although these phenomena are closely associated, patients with hyposalivation do not necessarily report xerostomia. On the other hand, even if the majority of patients with xerostomia actually suffer from hyposalivation, they occasionally have regular salivary flow [8]. Xerostomia is very frequent in both T1DM and T2DM diabetic patients and may be present in >50% of cases [5,9]. The dry mouth symptom is one of the most common oral complaints in patients with DM. Moreover, the prevalence of xerostomia and hyposalivation has been reported to increase with age [10]. Overall, there are many ways in which dry mouth influences the quality of life of the elderly [11]. People with decreased salivary flow experience symptoms that may include the burning and itching of the oral mucosa and tongue. They also have trouble chewing, tasting, and swallowing foods and communicating with others [12]. The aetiology of dry mouth is unclear, but hyperglycemia in patients with DM results in polyuria and osmotic diuresis, thus causing dehydration, which is related to a reduced salivary flow. In addition, some antidiabetic drugs (SGLT-2 inhibitor) inhibit the reabsorption of glucose in the kidneys and then glycosuria. These conditions entail dehydration and therefore oral dryness [13]. Moreover, many patients with DM suffer other complications related to this disease, for which they may receive drugs (e.g., anti-cholinergic and antihypertensive medication) which reduce the salivary flow and lead to xerostomia [14]. Numerous saliva substitutes have been produced to reduce oral dryness in the case of reduced salivary stimulation and when xerostomia persists [15,16]. It has been observed that it is possible to reduce xerostomia with the use of artificial saliva gel [16,17]. Moreover, according to Montaldo et al. in the case of hyposalivation in T2DM patients, a therapy with an immunologically active salivary substitute can also be used for reducing the amount of plaque and gingivitis [18].

Cornelli et al. previously studied the effects of aldiamed^®^ spray in type 2 DM patients after 3 days of use. However, the limits of this study were the small sample size (21 patients interviewed and only 16 included) and the short period of observation (only 3 days) [19]. Thus, to the authors best knowledge, this is the first clinical trial using aldiamed^®^ and a placebo in a randomized way for such a long period (30 days). In addition, the product is registered in the EU as a medical device and has already been successfully studied in a preliminary experiment in sleep apnea [20]. Starting from this consideration, the aim of this randomized double-blind controlled trial was to evaluate the clinical efficacy of an artificial saliva spray in patients with DM compared to a placebo spray through clinical and radiological analyses.

Moreover, there are different studies in the literature that have observed structural changes in the salivary glands of patients with DM, in particular in the parotid glands [21,22,23].

In our study, we decided to include, among the secondary outcomes, any changes related to the conditions of the salivary glands, because in the literature such phenomena as asymptomatic enlargements of the salivary glands might indicate a compensatory mechanism to counteract hyposalivation. Therefore, we decided to analyze also the structural modifications of the salivary glands (in cases where there were any) to better understand the phenomenon. The null hypothesis of the present study was that there were no differences in terms of xerostomia in aldiamed and the placebo after 30 days of treatment.

## 2. Experimental Section

### 2.1. Experimental Design

**Figure 1:**
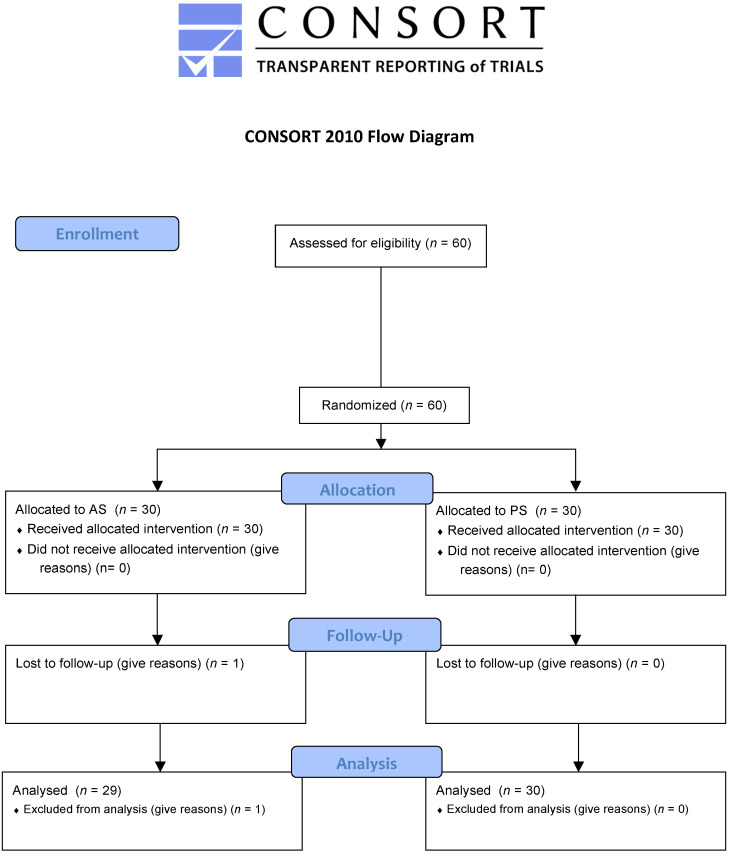
Consort 2010 flow diagram.

This double-blind, randomized, controlled clinical trial (Figure 1) was performed on 60 T1DM and T2DM patients. They were divided in a randomized way into two groups: the patients who were treated with the aldiamed^®^ spray (AS) test group and the patients treated with a placebo spray (PS) (flavored physiological solution). The main outcome was to evaluate the xerostomia at the baseline and after 30 days of treatment. The secondary outcomes were the evaluations of four parameters: simulated salivary flow rate, the antioxidant power of saliva, oral cavity conditions, and an ultrasound of the major salivary glands. Finally, laboratory tests (blood crasis, transaminases, bilirubin creatinine, etc.) were carried out to better understand the general tolerance to the aldiamed^®^ spray.

### 2.2. Study Population

From November 2018 to December 2019, a total of 60 patients (44 males and 16 females) aged between 25 and 55 years old (the mean average age for the AS group was 39 ± 4.3 years old and 40 ± 3.5 for the ps group) were recruited at the Department of Medical, Oral, and Biotechnological Sciences of the “G. D’Annunzio” University of Chieti-Pescara, Italy. The participants volunteered for the study after they were informed and asked to sign a consent form. Moreover, their relatives and family doctors were informed of their participation in this study. The study protocol was approved by the Ethical Committee of the “G. Annunzio University” of Chieti and Pescara: No. 14 of 8/11/2018. The eligibility criteria for the participants were: diagnosis of T1DM and T2DM of at least 1 year, and with a stabilized therapy for at least 6 months; glycated hemoglobin (HbA1c9) values of <7.5; current therapies for chronic diseases proved to be stable for at least the last 6 months; xerostomia a subjective scale score of >2. The evaluation of the antioxidant power of the saliva was not considered a limitation criterion for the admission in the present study. The exclusion criteria were: obesity (Body Mass Index >30 kg/m^2^); any type of tumor; alcoholism or drugs of any type (for the risk of xerostomia); depression; chronic/degenerative Central Nervous System diseases; and a xerostomia subjective scale score of <2. Moreover, patients who were in therapy with any type of drugs that cause xerostomia as side effect were not included in the present study. Salivary flow was not considered as an exclusion criterion.

### 2.3. Sample Size Calculation and Data Statistical Analysis

To calculate the sample size of the present study, the value of the primary outcome (variable) was taken in a previously published paper. In fact, the xerostomia data previously collected from 10 subjects indicated an average score value of 4.2 ± 0.75. Considering that a clinically valid average reduction in the same subjects could be equal to 3 ± 0.75 and that α = 0.05 and 1-β = 0.80, a sample of 11 cases would be enough to discriminate the two treatments. Moreover, considering the presence of 30% of dropouts, we decided to use a sample of 30 cases/group. [19]. 

### 2.4. Blinding Protocol (Double Blind Conditions)

**Figure 2:**
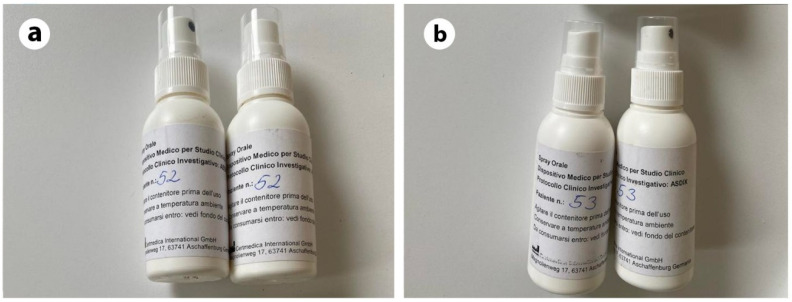
Spray bottles identical for the aldiamed^®^ spray (AS) and placebo spray (PS) groups (**a**,**b**).

The products were packaged anonymously and characterized by a progressive number (from 1 to 60). Patients were randomly divided into two groups, the AS group (test) and the PS group (control), as indicated by the randomization chart. The randomization was obtained using computer generated random numbers, centralized with sequentially sealed opaque envelopes provided by the study advisor as previously performed [24]. The envelope was opened only after the study was finished to reveal the allocation of the patients. Three spray bottles were provided to each patient containing an identification number. A double label was attached to each spray bottle and one of these was attached to the patient’s medical record. The randomization list was known only to an officer not participating in the trial or evaluating the data. Both the patient and scientific experts were “blind” for what concerns the group they were assigned to. In addition, the AS and PS packages were identical in appearance (Figure 2).

### 2.5. Analysis

#### 2.5.1. Variables Modifications during the Study

The 5 variables analyzed were: xerostomia (main variable), salivary flow (secondary variable), antioxidant power of saliva [25] (or SAT, secondary variable), oral cavity conditions (secondary variable), and the ultrasound of the major salivary glands (secondary variable).

#### 2.5.2. Xerostomia

**Figure 3:**
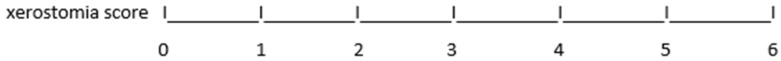
Visual Analogue Scale (VAS) of Xerostomia (0–6).

The main variable was xerostomia, and it was measured with a semi-quantal scale from 0 to 6 as follows (Figure 3):

The number “0” indicates mild oral dryness, while “6” is its maximum expression (intermediate scores of 0.5 are not contemplated, such as 1.5 or 2.5) and refers to the 24 h condition. This was indicated by each patient.

#### 2.5.3. Salivary Flow Rate 

The salivary flow rate was measured based on a previous study which used stimulated saliva sample collection by simply chewing a cotton square [25]. Briefly, before measuring the salivary flow rate, the empty plastic glass and the cotton square were measured and registered in the patient’s Case Report Form (CRF). Then, the patient was asked to chew the no flavoring cotton square, making 60 chewing motions in a minute and rolling the cotton in the mouth. Subsequently, the cotton was removed and inserted into a small plastic glass and weighed again, and the tare flow was obtained per minute. Under physiological conditions, this flow is >1 mL/min. The evaluation was always carried out in the morning. The patient was asked to not eat or drink before the examination either at the baseline or at the final visit.

#### 2.5.4. Salivary Antioxidant Capacity

The antioxidant activity of saliva was measured according to a Soluble Antioxidants Test (SAT) in terms of U. Cor/mL (Cornelli’s Units), corresponding to the mEq of ascorbic acid. For the evaluation, a photometer was used which measures the discoloration of a thiocyanate solution (SCN) that reacted with Fe^3+^ (ferric) ions to form Fe [(SCN)_6_]3^−^. The solutions containing SCN are characterized by a brown-red colored complex, and the presence of reducing agents reduces its intensity in a linear way [19].

#### 2.5.5. Oral Cavity Conditions 

The oral cavity conditions were also performed at the baseline and 30 days after treatment by the same dentist (B.S.) for all the participants as a variable to confirm the balance between the two groups, considering only the presence (score = 1) or the absence (score = 0) of the following signs and symptoms: burning mouth syndrome, candidosis, cavities, gingivitis/periodontitis, glossodynia, lichen planus, poor domiciliary oral hygiene care, and poor professional oral hygiene care (persons that have not performed a professional oral hygiene in the previous 6 months). Suggestions were given to the subjects about oral hygiene, such as tooth brushing at least twice daily (in the morning and before bed) for at least three minutes. All the data were recorded to the patient’s specific case report form.

#### 2.5.6. Ultrasonography of Salivary Glands

Ultrasonographic images of the main salivary glands (submandibular and parotid) were taken at the baseline and after 30 days of treatment. The scores for the dimension, structure, vascular analysis, presence of nodules, ducts characteristics, and lymph nodes were recorded. The score ranges simply from 0 to 1, where 0 represents normality and 1 represents any type of modification. 

### 2.6. Laboratory Analysis for Safety

In patients, the general tolerability parameters (red/white cells and platelets, transaminases (GOT, GPT), and bilirubin (direct and total creatinine)) were evaluated at the baseline and after 30 days of treatment. Blood glucose was not considered as a variable, since all the subjects were on insulin or oral antidiabetic therapy. Blood samples were collected in the morning from subjects fasting since the night before. Three aliquots of 5 mL of blood each (two in test tubes with sodium citrate and one without) were drawn from the brachial vein. The samples were placed in containers with dry ice and immediately dispatched to the analytical laboratory for the evaluation. These laboratory investigations were performed at the “Marchegiani Analysis Laboratory” in Pescara under the responsibility of Dr. Paola Marchegiani after blood sampling.

### 2.7. Settings, Study Procedures, and Interventions

Two types of treatment were analyzed: an artificial saliva spray (aldiamed^®^ spray, Certmedica International GmbH, Aschaffenburg, Germany) compared to a placebo spray (flavored physiological solution). Both would be administered at least 5 times a day. The product, aldiamed^®^ spray, is marketed in the EU as a medical device. Sufficient product packaging was distributed to each subject for the whole of the experimental experience. The survey involved the use of at least 5 administrations/day and a maximum of 8 administrations/day. Each administration consisted of 3 puffs, each of which has a volume of 0.12 mL. Therefore, a total of 0.36 mL would be administered. Taking into consideration that the maximum number of administrations was anticipated per day to be 8, the amount used should not exceed 3 mL/day. The aldiamed^®^ spray composition is reported in Table 1.

It was observed that the maximum daily quantities administered (up to 6 puffs) were extremely low; given the current use of the same active ingredients in many formulations for oral use in the free market, no toxicological effects were expected. The compliance was evaluated by considering the number of residual packages (and their weight). All the data were reported on a specific CRF for each patient. The data of all the patients were transferred to a single file to allow for statistical evaluation. In addition to the general and anamnestic characteristics, the folder also contained the variables measured at baseline and after 30 days of treatment (maximum 33 days). The patients were identified with a progressive number from 1 to 60. All the data were stored in the experimental center and made available upon reasonable request only.

### 2.8. Data Processing

The position and dispersion parameters were calculated for all the data. The ANOVA according to a split-plot design was applied to determine the differences between the two groups. A chi square with the Yates correction or a Fisher exact chi square was applied for the analysis of the frequencies.

## 3. Results

### 3.1. Study Population

After 30 days, 59 patients completed the trial. There was only one dropout in the AS group, and the withdrawal was due to logistic reasons. No side effects were reported for the entire period of the study for both the sprys used. However, there were three patients belonging to the PS group complaining of taste alteration (one patient) and gastric discomfort (two patients). Nevertheless, the symptoms disappeared without any treatment, and all the participants completed the trial. Due to the limited number of females in the two groups (seven in the AS group and eight in the PS group), it was not possible to analyse the effect of gender differences. The number of puffs/day was almost identical in both treatments, not exceeding 6 puffs/day (between five and six). The subjects’ characteristics are reported in Table 2.

### 3.2. Principal Findings 

The average scores of xerostomia at baseline were similar in both the treatments (*p* > 0.05). The two treatments improved the symptoms significantly (U Mann Whitney test *p* < 0.05), but the AS was found to be significantly more effective than the PS (ANOVA). The salivary flow rate was not modified in any of the two groups (t test *p* > 0.05). The increase in SAT was +11% for the AS group and 15% for the PS group; in both cases, the *p*-value was not statistically significant (t test *p* > 0.05). The results of the xerostomia, salivary flow rate, and antioxidant capacity of saliva are reported in Table 3. The general condition of the major salivary glands was physiological in most of the cases, apart from some minor modifications, which were well balanced in both groups. No statistically significant difference before and after the treatment between the two groups for any of the variables was shown (Fisher test *p* > 0.05). In some cases, the AS group showed improvements in the ultrasonographic images, but the differences with the PS group were not statistically significant (Fisher test). The ultrasound variables, such as dimension; structure; vascular analysis; nodules; and the ducts and lymph nodes of both the submandibular gland and parotid, right (dx) and left (snx) glands, reported no statistically significant differences between the two groups. Moreover, the data of the laboratory analysis for safety (Table 4) reported no statistically significant differences at baseline and after 30 days between the two examined groups.

## 4. Discussion

The null hypothesis under test was rejected, demonstrating that there was a statistically significant difference in terms of xerostomia between the two groups. T1DM and T2DM are known to very frequently reduce the salivary flow rate and are probably the cause of subjective xerostomia [26]. It was decided to include in this study both type I and II diabetic patients, because no differences were reported in the literature in terms of xerostomia between these two types of Diabetes Mellitus [9]. Additionally, the study design was based on a previously published paper which reported no obvious difference in the salivary flow rate between type I and II diabetic patients and healthy individuals [27]. As reported in Table 2, the T1DM for the AS group were 9/29 patients and 10/30 for the PS group. Meanwhile, T2DM were 20/29 and 20/30 specifically for the aldiamed and placebo groups. In particular, when there is dehydration and an inadequate control of hyperglycaemia, this disease may increase the susceptibility to tooth caries and the tendency for infections [28]. This is the reason why oral hygiene is the key tool for an effective reduction in xerostomia, followed by artificial saliva use and, if necessary, treatment with drugs (e.g., anticholinergics), which may stimulate and increase the salivary flow rate [29,30]. It should be noted that the saliva’s antioxidant capacity has not improved in the current study, since 50% of participants were affected by gingivitis or periodontitis in both groups, as shown in Table 2. Moreover, the association between periodontitis and diabetes has been widely validated in the literature, with a reported bidirectional influence ratio [31]. According to Loe et al. [32], periodontitis represents the sixth complication of patients with diabetes, and it has also been reported that people with periodontitis are more susceptible to developing this disease [33]. It has been reported that most patients with DM have salivary dysfunction as well as alterations in biochemical and microbiological salivary composition compared to healthy subjects [34], and this entails a high incidence of dental caries [35]. This is also reported by our data, which show that more than half of the participants presented dental caries to be treated. All the patients were advised that the caries should be treated to improve their oral health. The examinations regarding the dental caries, periodontal health, and oral cavity status were carried out by the same dentist. Only a very few patients were on drug treatment for chronic conditions other than insulin or oral hypoglycaemic drugs, but these drugs do not influence the salivary flow rate or other patient conditions. The average stimulated salivary flow in both groups was >2 mL/min, indicating that for most of the cases in this experiment, xerostomia was to be considered subjective. The daily use of 5 to 6 puffs of aldiamed^®^ spray was significantly more effective than the same quantity of placebo, most probably due to the more consistent permanence of fluidity in the oral cavity. The combination of components such as aloe vera, xylitol, and propylene glycol in the product is aimed at maintaining an appropriate humidity of the oral mucosa. In fact, previously it was demonstrated that aldiamed^®^ spray can improve the structural softness, used as a measure of lubricity, of existing salivary condition films [15]. Our results highlighted the effects of this spray, as previously reported, on reducing the xerostomia to T1DM and T2DM patients. Momm et al. in 2005 tested the use of four saliva substitutes for the treatment of xerostomia in a prospective crossover study. Specifically, they administered questionnaires demonstrating the perception of a reduction in xerostomia by the enrolled patients. Our results did not demonstrate statistically significant differences between the test and placebo, measured by objective methods [36]. Moreover, previous findings showed that patients with DM had a higher prevalence of xerostomia and a lower salivary flow rate than non-DM patients [37]. In fact, a non-statistically significant difference in terms of saliva flow rate or antioxidant capacity was shown after one month of treatment between the two groups. This time point was chosen based on a previous study by Femiano et al. 2011 [38]. The modification of the salivary flow rate was not expected, since local treatments with nonpharmacological agents cannot modify saliva production by the major glands, such as the submandibular or parotid glands. Similarly, the antioxidant capacity of the oral cavity was not modified, probably because it is directly dependent on the increase in saliva production. The absence of differences between the placebo and the aldiamed might be due to the short period of observation. Thus, it would be necessary to further analyze the effects of these two sprays in a longer period in the future. Our results are in accordance with Villa et al. [39], who have shown that there is no reliable evidence for any saliva substitute to relieve the symptoms of dry mouth in terms of increased salivary flow rate and the modification of its components [39]. These findings are related to the methods currently applied to compare and validate saliva substitutes, including visual analogues in vivo scale, index and questionnaires, and the (non) stimulated measurement of the salivary flow [38,40]. In the light of these methods, there is no reliable technique to objectively measure the lubricating properties of salivary substitutes.

On the other hand, the ultrasonography of the major salivary glands was compromised in only a very few subjects, and the treatment was not expected to allow consistent modifications of the glandular function/structure due to the short period of time. In fact, there are few studies that have observed structural changes in the salivary glands of patients with DM, due to compensatory mechanisms against hyposalivation or xerostomia [41]. Moreover, this has been observed in the submandibular gland of patients with T2DM enlargement of the acini, even in the absence of impaired glandular function [22]. The substitute for artificial saliva may be a valid treatment to relieve the subjective disorders of xerostomia, since they should contain thickeners that increase the stability of the liquid for long-lasting relief and allow a good moistening of the oral cavity. An ideal salivary substitute should be pleasantly flavoured, biocompatible, and inexpensive, and must have a favourable wetting capacity on oral surfaces [42]. In our case, aldiamed^®^ spray containing aloe vera helps to protect oral tissues with healing properties, antibacterial activity, and moisturizing effects [43]. Several studies have been carried out on aloe vera, demonstrating its antioxidant and anti-inflammatory capacities. Moreover, it also has antibacterial activity. It should be remembered that the acemannan contained in aloe vera has properties which can be useful for oral health [44,45,46]. In addition, the aldiamed spray used in the present trial had as one of its components aloe vera. In this case, the aldiamed^®^ spray was found not to compromise the common variables mirroring the toxic effects. Some limitations of the present study must be taken into consideration—first of all, the short period of observation, which was only 30 days. Surely, it would be interesting to observe the same parameters after a longer study period than 30 days and through a crossover study. Moreover, other variables, such as the oral health-related quality of life, should be further investigated.

## 5. Conclusions

The use of Aldiamed^®^ spray was shown to be more effective than a placebo in the treatment of xerostomia in type 1 and type 2 diabetes, confirming the data of a previous short-term study [19]. It seems that the effect is due to the relief of subjective xerostomia. It should be taken into consideration that placebo effects are psychobiological phenomena that can be relevant in both laboratory and clinical settings [47]. However, long-term studies are needed to determine the benefit of the product, in terms of decreasing the effects of the xerostomia or decreasing the salivary flow rate in the oral cavity.

## Figures and Tables

**Table 1:** Aldiamed^®^ spray main component/puff.

Component	%	Quantity (× puff mg)	Quantity (× 3 mL mg)
Water	70	0.12	2.88
Xylitol	10	0.084	2.02
Propylene glycol	10	0.012	0.29
Other (Aloe Vera, Lysozyme, Lactoferrin)	10	0.012	0.288
Total	100	0.12	2.88

**Table 2:** Subjects characteristics: frequency of total cases or ranges with the relative statistical significance.

Variable	AS	PS	P
Sex M/F	22M/7F	22M/8F	Ns
Age, years	>25, <55	>25, <55	Ns
BMI	>23, <26	>23, <26	Ns
Type 1 Diabetes	9/29	10/30	Ns
Type 2 Diabetes	20/29	20/30	Ns
Other chronic treatments	3/29	4/30	Ns
Smoking	1/29	2/30	Ns
Burning mouth syndrome	1/29	1/30	Ns
Candidosis	1/29	1/30	Ns
Tooth caries (treated and non-treated)	20/29	19/30	Ns
Gingivitis/periodontitis	15/29	15/30	Ns
Glossodynia	2/29	2/30	Ns
Lichen planus	0/29	1/30	Ns
Poor domiciliary oral hygiene care	0/29	1/30	Ns
Poor professional oral hygiene care	13/29	14/29	Ns

AS = aldiamed^®^ spray; PS = placebo spray; P = *p*-value of anova test; Ns = not statistically significant.

**Table 3:** Xerostomia score, salivary flow, saliva antioxidant capacity (SAT): mean values ± SD at baseline and after 30 days of treatment, with the relative statistical significance (pa) for each variable between the two groups.

Variable	Measure	AS		PS		Pa	
		Baseline (B)	30 Days (30)	Baseline (B)	30 Days (30)	BVS B	30VS30
Xerostomia	score	2.9 ± 1.31	1.4 ± 1.48	3.0 ± 1.44	2.4 ± 0.99	Ns	<0.01
Salivary flow	mL/min	2.4 ± 1.06	2.7 ± 0.71	2.5 ± 0.75	2.9 ± 0.83	Ns	Ns
SAT	U.Cor/mL	1717 ± 867.6	1906 ± 1424.9	1361 ± 589.8	1570 ± 930.9	Ns	Ns

U.Cor = Cornelli’s Units, AS = aldiamed^®^ spray; PS = placebo spray; Pa = *p*-value of anova test; Ns = not statistically significant. (*p* > 0.05); BVSB = baseline versus baseline; 30VS30 = 30 days versus 30 days; SAT = saliva antioxidant capacity.

**Table 4:** Laboratory analysis: mean ± SD at baseline and after 30 days.

Variable	Measure	AS		PS		Pa	
		Baseline	30 Days	Baseline	30 Days	Baseline	30 Days
White cells	103	6.25 ± 1.787	6.25 ± 1.828	6.86 ± 1.288	6.97 ± 1.611	Ns	Ns
Red cells	106	4.77 ± 1.023	4.76 ± 0.986	5.05 ± 0.526	5.01 ± 0.614	Ns	Ns
Platelets	103	208 ± 73.6	209 ± 64.5	221 ± 81.6	224 ± 70.8	Ns	Ns
Hemoglobin	g/100 mL	14.2 ± 2.94	14.3 ± 2.96	14.6 ± 1.27	14.5 ± 1.51	Ns	Ns
Hematocrit	%	41.9 ± 8.44	41.2 ± 8.36	43.5 ± 3.03	42.3 ± 4.02	Ns	Ns
Creatinine	mg/dL	1.05 ± 0.232	0.97 ± 0.228	1.03 ± 0.157	0.95 ± 0.138	Ns	Ns
GOT	UI	25.0 ± 14.5	25.1 ± 12.10	22.2 ± 5.98	23.9 ± 5.90	Ns	Ns
GPT	UI	28.9 ± 20.73	28.7 ± 17.08	25.8 ± 13.95	27.7 ± 16.93	Ns	Ns
Bilirubin (direct)	mg/dL	0.28 ± 0.098	0.29 ± 0.092	0.28 ± 0.076	0.27 ± 0.042	Ns	Ns
Bilirubin (total)	mg/dL	0.85 ± 0.430	0.83 ± 0.427	0.79 ± 0.297	0.75 ± 0.229	Ns	Ns

AS = aldiamed^®^ spray; PS = placebo spray; Pa = *p*-value of anova test; Ns = not statistically significant. (*p* > 0.05); GOT = Glutamic Oxaloacetic Transaminase; GPT = glutamic-pyruvic transaminase.
